# *Paracoccidioides* spp.: the structural characterization of extracellular matrix, expression of glucan synthesis and associated genes and adhesins during biofilm formation

**DOI:** 10.3389/fmicb.2024.1354140

**Published:** 2024-03-07

**Authors:** Lariane Teodoro Oliveira, Caroline Maria Marcos, Ana Karla Lima Freire Cabral, Kaila Petronila Medina-Alarcón, Regina Helena Pires, Ana Marisa Fusco-Almeida, Maria José Soares Mendes-Giannini

**Affiliations:** ^1^Department of Clinical Analysis, School of Pharmaceutical Sciences, São Paulo State University (UNESP), Araraquara, Brazil; ^2^Laboratory of Medical Mycology, School of Pharmaceutical Sciences, Federal University of Amazonas-UFAM, Manaus, Brazil; ^3^Laboratory of Mycology and Environmental Diagnosis, University of Franca, Franca, Brazil

**Keywords:** *Paracoccidioides* spp., biofilm, extracellular matrix, qRT-PCR, glucans, adhesins

## Abstract

The genus *Paracoccidioides* includes *Paracoccidioides lutzii* and the *Paracoccidioides brasiliensis* complex, which comprises four phylogenetic species. A key feature distinguishing planktonic growth from biofilm is the presence of a 3D extracellular matrix (ECM). Therefore, in this study, we analyzed biofilm formation in different species of *Paracoccidioides* yeast phase, characterized the structural elements of the matrix of *P. brasiliensis* (Pb18), *P. lutzii* (Pl01 and 8334) and *P. restrepiensis* (339 and 192) and evaluated the expression of glucan genes, according to the stage of biofilm evolution for *P. brasiliensis*. The strains were cultivated in planktonic and biofilm form for 24–144 h. The fungi biomass and metabolic activity were determined by crystal violet and tetrazolium salt reduction (XTT) tests and colony-forming unit (CFU) by plating. The biofilm structure was designed using scanning electron microscopy and confocal laser scanning microscopy techniques. The extracellular matrix of *P. brasiliensis* and *P. lutzii* biofilms was extracted by sonication, and polysaccharides, proteins, and extracellular DNA (eDNA) were quantified. The RNA was extracted with the Trizol^®^ reagent and quantified; then, the cDNA was synthesized to analyze the enolase expression, 14-3-3, FKS1, AGS1, GEL3, and KRE6 genes by real-time PCR. All strains of *Paracoccidioides* studied form a biofilm with more significant metabolic activity and biomass values in 144 h. The extracellular matrix of *P. brasiliensis* and *P. lutzii* had a higher content of polysaccharides in their composition, followed by proteins and eDNA in smaller quantities. The *P. brasiliensis* biofilm kinetics of formation showed greater expression of genes related to glucan's synthesis and its delivery to the external environment in addition adhesins during the biofilm's adhesion, initiation, and maturation. The GEL3 and enolase genes increased in expression within 24 h and during the biofilm maturation period, there was an increase in 14-3-3, AGS1, and FKS1. Furthermore, at 144 h, there was a decrease in KRE6 expression and an increase in GEL3. This study highlights the potential for biofilm formation for three species of *Paracoccidioides* and the main components of the extracellular matrix that can contribute to a better understanding of biofilm organization.

## Introduction

Paracoccidioidomycosis (PCM) is a systemic mycosis that assumes clinical importance due to increased prevalence and mortality rates. PCM was included on the World Health Organization (WHO) Fungal Priority Pathogens List due to their endemicity in the Americas, high morbidity, and potential for sequelae that negatively impact the quality of life (WHO, 2022). The genus *Paracoccidioides* includes *Paracoccidioides lutzii* and the *Paracoccidioides brasiliensis* complex, which comprises four phylogenetic species, including *Paracoccidioides brasiliensis* (S1a and S1b), *Paracoccidioides americana* (PS2), *Paracoccidioides restrepiensis* (PS3), and *Paracoccidioides venezuelensis* (PS4) (Turissini et al., 2017; Teixeira et al., 2020). PCM affects the human lungs and can progress to systemic granulomatous disease with tegumentary and visceral involvement (de Oliveira et al., 2015; Hahn et al., 2022).

The pathogenicity of fungi in humans depends on various virulence factors. Dimorphism, cell wall composition, production of metalloproteins, adhesins, melanin, and indirectly the formation of biofilm has been reported as virulence factors of the genus *Paracoccidioides* (Mendes-Giannini et al., 2008; Sardi et al., 2015; Scorzoni et al., 2015; Camacho and Niño-Vega, 2017; Santos et al., 2020). The presence and content of α-glucans in the cell wall of yeast forms of *P. brasiliensis* was related to their virulence (San-Blas and Niño-Vega, 2008). Proteins such as 14-3-3 and enolase act as adhesins, playing an important role during *Paracoccidioides* spp. infection. A previous study showed a correlation between the expression of adhesins and virulence since the blockade of 14-3-3 and enolase adhesins resulted in reduced adhesion of *Paracoccidioides* spp. to lung cells of the A549 lineage (de Oliveira et al., 2015). Additionally, when adhesins were blocked, the survival rate was higher in mice (C57BL/6) and *Galleria mellonella* larvae (de Oliveira et al., 2015).

The biofilm formation by *P. brasiliensis* was first reported by Sardi et al. (2015) in abiotic surfaces (polystyrene plate) under hypoxic conditions. Also, the fungal agglomeration resembling biofilm was observed when interacting with epithelial and macrophage cells, an aspect that can be considered similar to that found in granulomas, which consists of a nodular arrangement in a hypoxic environment, containing many types of cells, as epithelioid and Langhans cells, and also yeasts (Fortes et al., 2011; Grahl et al., 2012). The gene expression at mature biofilm of *P. brasiliensis* demonstrates higher expression of gp43 and GAPDH known to be adhesins in *P. brasiliensis*, as well as up-regulation of aspartyl proteinase gene that can contribute to invasion and tissue destruction as described for *Candida albicans* (Vicentini et al., 1997; Hanna et al., 2000; Barbosa et al., 2006; Ramage et al., 2012; Sardi et al., 2015).

Posteriorly, Cattana et al. (2017) reported the presence of a dense network of *Paracoccidioides* yeast cells held by a dense ECM, observed by scanning electron microscope, forming a biofilm at a vascular prosthesis in a patient that's suffered intestinal infarct and acute ischemia in the right lower limb. Therefore, this prosthesis was removed for analysis, being this the first description of *in vivo* biofilm formation of *P. brasiliensis*.

The ECM can trigger higher resistance to antimicrobials, mainly due to the difficulty of their diffusion through the matrix, making the treatment of infections related to the biofilm challenging (Chandra et al., 2001; Mitchell et al., 2015; Silva et al., 2017; Oshiro et al., 2019).

The “matrixome” is the inventory of currently known biomolecules (polysaccharides, nucleic acids, proteins, lipids, and lipoproteins), their molecular, structural, and functional diversity associated with biofilm assembly, and their physicochemical and virulence attributes. The biofilm properties are related to surface adhesion, spatial and chemical heterogeneity, synergistic/competitive polymicrobial interactions, antimicrobial recalcitrance, and biofilm virulence (Karygianni et al., 2020).

The ECM corresponds to 70–95% of the organic matter of the biofilm mass, of heterogeneous and complex composition composed of four classes of macromolecular: proteins, carbohydrates, lipids, and nucleic acid (Flemming et al., 2000; Mitchell et al., 2016; Bamford et al., 2020). Studies on the matrix have reported that it is assembled in the extracellular environment, from the incorporation of a variety of products from the community to create a unique structure (Sutherland, 2001; Garny et al., 2010; Mitchell et al., 2015; Desmond et al., 2018; Zarnowski et al., 2018).

The composition of the *Paracoccidioides* extracellular matrix has not yet been characterized. Polysaccharides are essential in most biofilm matrices (Flemming et al., 2007), representing ~25% of their dry weight (Zarnowski et al., 2014). Alpha-mannan and β-1,6 glucan comprise 85 and 14%, respectively, of *C. albicans'* matrix carbohydrates, with β-glucan secretion in its biofilms regulated by gene expression in response to quorum sensing (Mitchell et al., 2015) and the increased expression of β-glucans in the matrix implies the sequestration of antifungal agents, reducing the susceptibility of *C. albicans* biofilms (Nett et al., 2010a; Zarnowski et al., 2018). Several glucan and mannan synthase and modification genes in *C. albicans* were important for producing the extracellular matrix components (Nett et al., 2010a,b; Taff et al., 2012; Mitchell et al., 2015).

Another constituent of the biofilm matrix is extracellular deoxyribonucleic acid (eDNA) found in the extracellular medium. The mechanism by which eDNA is secreted into the biofilm microenvironment is not fully understood. For bacteria, some mechanisms have been proposed, such as cell lysis and secretion via vesicles (Allesen-Holm et al., 2006) and for *C. albicans* and *Aspergillus fumigatus* (Rajendran et al., 2014, 2016), maybe cell lysis is involved in this process. In addition, the accumulation of toxic metabolites and the scarcity of nutrients resulting from limited diffusion by the matrix favors the expression of enzymes that remodel the cell wall, releasing nutrients to the microbial community, such as chitinases, which, when degrading chitin of the cell wall, cause DNA leakage to the outside environment (Rajendran et al., 2014). A previous study reports that chitin expression in the mature biofilm is increased and that chitinase inhibition alters the growth and stability of *A. fumigatus* and *C. albicans* biofilms (Rajendran et al., 2014).

Thus, in this study, we intended to analyze biofilm formation in different species of *Paracoccidioides*, characterize the structural elements of the matrix of *Paracoccidioides* spp., and describe the differential expression of genes related to glucan's synthesis and its delivery to the external environment and adhesins during the adhesion, initiation, and maturation of the *P. brasiliensis* biofilm. These factors may be necessary to understand biofilm formation better, giving insights into the determination of targets to be genetically modified to elucidate its role and, in the future, contribute to the development of anti-biofilm therapies.

## Materials and methods

### Microorganisms

The strains *P. brasiliensis* (Pb18), *P. lutzii* (Pl01 and 8334), and *P. restrepiensis* (339 and 192) were used in the yeast phase, obtained from the Mycology Laboratory of the Department of Clinical Analysis of the Faculty of Pharmaceutical Sciences, Campus of Araraquara, UNESP. The strains were cultivated in solid Fava-Netto medium at 37°C for 5 days (Restrepo et al., 2015).

### *Paracoccidioides* spp. growth conditions and *in vitro* biofilm formation

The *Paracoccidioides* spp. strains were cultured in Fava-Netto medium for 5 days at 37°C. We followed the biofilm growth condition described by Oliveira et al. (2020). Briefly, a suspension containing 1 × 10^6^ cells/mL in Brain Heart Infusion (BHI) medium supplemented with 1% of glucose at a higher viability condition, >90%, evaluated with Trypan blue assay, was plated on a polystyrene 24-well microtiter plate, adding 1 mL of inoculum. The plates were incubated at 37°C without agitation for 24, 48, 96, 144, and 168 h (two plates each time), changing the medium every 24 h. Concomitantly to the planktonic growth conditions, the same initial inoculum was seeded in 200 mL of BHI plus 1% glucose at the same final concentration and incubated at 37°C under the agitation of 150 rpm and at the periods of 24, 48, 96, 144, and 168 h. An aliquot of 48 mL was taken for RNA extraction.

### Quantitative analyses of monospecies and mixed biofilms

#### Total biomass quantification

The quantification of the total biomass of biofilms was determined based on the ability of crystal violet to penetrate the cell wall of microorganisms and remain retained in the cytoplasm. After the growth of biofilms in microplates (96 wells), the supernatant from each well was aspirated and washed twice with PBS. Two hundred microliter of methanol was added per well, which was maintained for 15 min for fixation and then aspirated and dried for 45 min. After 200 μL of 0.1% crystal violet (CV) for 20 min was added. After washing with distilled water to remove excess, the CV was solubilized with 200 μL of 33% acetic acid. After 10 min, the absorbance was read at 570 nm in a microplate reader (Sherry et al., 2014).

#### Quantification of metabolic activity

The kinetic of monospecies biofilm formation for *P. brasiliensis, P. lutzii*, and *P. restrepiensis* was obtained using the tetrazolium salt reduction test—XTT [2,3-Bis-(2-Methoxy-4-Nitro-5-Sulfophenyl)-2H-Tetrazolium-5-Carboxanilide], The reduction of XTT is used to measure the cellular viability of fungal biofilms by evaluating the metabolic activity, which promotes an intracellular reduction of salt forming a compound called formazan, which can be quantified by the color change (orange). A solution of XTT salt (0.1 mg/mL in PBS) and menadione (1 mM in ethanol; Sigma Aldrich, São Paulo, SP, Brazil) was prepared for these assays. A 100 μL aliquot of menadione XTT was then added to both prewashed biofilms and controls. The plates were incubated in the dark for 3 h at 37°C, and the colorimetric change was measured in a microplate reader at 492 nm. Wells containing culture medium/biofilms/XTT/menadione (positive control) and wells containing culture medium/XTT/menadione (negative control) were included (Martinez and Casadevall, 2007; Silva et al., 2010). The measurement is carried out at defined time intervals, providing data for constructing a kinetic curve of biofilm formation.

#### Quantification of colony forming units (CFU/mL)

After biofilm formation, at different incubation times (0, 24, 48, 72, 96, 120, 144, and 168 h), the wells were washed thrice with PBS (pH 7.2–7.4) to remove non-adherent cells. Then, 100 μL of BHI broth was added to each well; biofilms were scraped off, and the well of plate contents were individually transferred to microtubes containing 900 μL of BHI broth. Biofilms were mechanically disrupted by vigorous agitation for 30 s on a vortex, serially diluted, and plated on BHI agar supplemented with 1% glucose, 5% *P. brasiliensis* culture filtrate, and 4% fetal bovine serum to obtain cell counts of *Paracoccidioides* spp. in monospecies biofilms.

### Determination of the structure of monospecies biofilms

#### Confocal laser scanning microscopy of monospecies biofilms of *Paracoccidioides* spp.

In this step, only the strains of *P. brasiliensis* (Pb18) and *P. lutzii* (Pl01) were employed due to the clinical importance and incidence of these species. After the growth of biofilms in 24-well plates, the supernatant from each well was aspirated, and the biofilms were washed three times with PBS (pH 7.2–7.4). The first labeling was performed with fluorescein isothiocyanate (FITC—Fluorescein Isothiocyanate) showing the fungal cell, adding 500 μL of FITC (100 μg/mL, Invitrogen, Life Technologies, Brazil) per well for 30 min at room temperature. After incubation, washing was performed twice with PBS, and 500 μL of SYPRO Ruby (Invitrogen, Molecular Probes, Eugene, OR) was added for 20 min, evidencing the protein content of the biofilm matrix. After washing twice with PBS (pH 7.2–7.4), 500 μL of HOECHST (10 mM, Invitrogen, Life Technologies, USA) was added for 30 min to mark the nucleic content. After incubation, the biofilms were washed with PBS, and 1 mL of PBS (pH 7.2–7.4) was added for analysis. Analyzes were performed using a laser scanning confocal microscope (Carls Zeiss LSM 800, Germany). Each field of view was imaged using the 405 nm laser to capture HOECHST with an emission range of up to 450 nm, 488 nm for FITC detection with an emission range of up to 540 nm, and 280 nm for SYPRO Ruby with a range of emission up to 450 nm. Images were acquired using Image J 1.51p software.

### Analysis of the composition of the extracellular matrix of monospecies biofilms of *Paracoccidioides* spp.

#### Extraction of the extracellular matrix

Biofilms of *P. brasiliensis* and *P. lutzii* monospecies were subjected to matrix extraction by sonication with modifications, as previously described by Martins et al. (2010). Briefly, after biofilm formation in 24-well plates, biofilms were collected and transferred to conical tubes, resuspended in 10 mL of sterile water, and vortexed for 1 min. They were then sonicated for 20 min, followed by a 2-min vortex step, and centrifuged at 1,500 × g for 20 min. The supernatant was recovered, and then the analyses were performed.

#### Determination of total polysaccharide content

The total biofilm and matrix polysaccharide content was determined following the protocol described by DuBois et al. (1956). After extracting the extracellular matrix, 0.5 mL of the extract was used, and 12.5 μL of a phenol solution (80%) was added, followed by 1.25 mL of concentrated sulfuric acid and mixed. The samples were left to rest for 10 min and incubated in a water bath at 30°C for 20 min, with the absorption measured at 485 nm in a spectrophotometer. The polysaccharide content was calculated using glucose to obtain the standard curve (0–200 μg/mL).

#### Protein content

Protein content was quantified using Bradford's (1976) method. Briefly, after matrix extraction, 5 μL of samples were placed in 250 μL of Bradford reagent (BioRad) and incubated in the dark at room temperature for 10 min. After incubation, the reading was taken at 595 nm in the spectrophotometer. Protein content was calculated using bovine serum albumin (BSA) for the standard curve.

#### eDNA dosage

The eDNA was extracted as previously described (Martins et al., 2010) with some modifications. Briefly, phenol:chloroform: isoamyl alcohol (25:24:1, v/v) was added to the planktonic cell, biofilm, and extracellular matrix samples, vortexed and centrifuged at 1,732 × g for 20 min (Sorvall X1R, Thermo Scientific, West Chester, PA, USA). The supernatant was transferred to another tube, and ice-cold isopropanol (1:1) was added for DNA precipitation, followed by incubation for 1 h on ice. After centrifugation at 10,000 × g; 15 min at 4°C, the pellet was washed with ice-cold 70% ethanol, air-dried, and resuspended in 50 μL Tris-HCl EDTA buffer (TE). The eDNA concentration was measured by determining the absorbance at 260 nm in a spectrophotometer.

### Genomic analysis

#### RNA extraction

After incubation of pre-determined cultivation periods to biofilm condition, the supernatant was removed, followed by gentle washing with PBS to remove non-adherent cells. The same was collected after adding 750 μL of Trizol^®^ reagent (Ambion Life Technology) per plate and with a mechanical detachment with a rubber scrapper. To planktonic condition, the aliquots were centrifuged at 3,000 rpm for 5 min, the supernatant was discarded, and the cell pellets were resuspended with three volumes of Trizol^®^ reagent. The RNA extraction was performed as described by the manufacturer's instructions with some modifications, including cell disruption with glass beads, vigorous vortexing, and intercalating with ice baths. The total RNAs were quantified by spectrophotometry and their integrity was assessed by 0.8% agarose gel electrophoresis. Aliquots of extracted RNA were stored at −80°C until the use.

#### cDNA synthesis

At first, genomic DNA from 1 μg of total RNA was removed with DNAse (Thermo Fisher Scientific) according to the manufacturer's instructions. Secondly, cDNA was synthesized using RevertAID^TM^ H Minus Reverse Transcriptase (Fermentas). The relative expression levels of enolase, 14-3-3, FKS1 (1,3-beta-glucan synthase), AGS1 (1,3-alpha-glucan synthase), GEL3 (1,3-beta glucanosyltransferase) and KRE6 (glycosyl hydrolase) genes were analyzed. ACT1 (actin-1) and L34 (ribosomal 60S subunit) were compared to the use as endogenous control genes, and the most adequate used the analysis (Goes et al., 2014; de Curcio, 2018). Real-time polymerase chain reactions (qRT-PCR) were performed in 20 μL reaction volumes, containing 1 × QuantiNOVA SYBR Green (Qiagen) master mix, 0.1 μL of Rox, cDNA templates (following four 10-fold serial dilutions: 10 ng, 1 ng, 100 pg, and 10 pg), 0.7 μmol of each primer pairs (Table 1) to generate a standard curve for each pair of primers. Control reactions were performed without template DNA, and cDNA was synthesized without the DNAse treatment. The real-time PCR amplification conditions were as follows: 95°C for 1 min for enzyme activation, a pre-denaturation step at 95°C for 1 min, followed by 40 cycles of 95°C for 5 s and 60°C for 32 s at Applied Biosystems 7500 cycler (Applied Biosystems). All melting curves were analyzed to confirm a single product that was also evaluated by electrophoresis. The efficiency (E) of the qPCR amplification was calculated using the slope value derived from the standard curve through the formula E = (10^−1/*slope*^ – 1) × 100. Data were analyzed using the 2^−Δ*ΔCt*^ method, where the Ct is the threshold cycle (Livak and Schmittgen, 2001).

**Table 1:** Gene information and oligonucleotides used in qRT-PCR.

**Oligonucleotide**	**Sequence (5^′^→3^′^)**	**Gene identification**	**Fragment lenght**	**References**
L34	**F:** TCAATCTCTCCCGCGAATCC	PADG_04085	118 bp	Goes et al., 2014
	**R:** AGTTGGCGATTGTTGTGCGG			
ACT1	**F:** CGTCCTCGCCATCATGGTAT	AY383732	142 bp	de Curcio, 2018
	**R:** TCTCCATATCATCCCAGTTCG			
ENOLASE	**F:** CCTACCGTTGAGGTTGATGT	EF558735.1	158 bp	This study
	**R:** TTGACGTTCTTGACTGCGTTC			
14-3-3	**F:** TGCCTCGTGGAGAATCGTTACT	AY462124.1	146 bp	This study
	**R:** TGTCAAGCACATCCAAGATATCCTCA			
FKS1	**F:** ATCAAGGGGCTGCAGGCTATCA	PADG_11846	169 bp	This study
	**R:** TTGTAGCTGTTGTACCGCTCGT			
AGS1	**F:** AAATGCGGCACGGAGGAGA	PADG_03169	149 bp	Navarro et al., 2021
	**R:** AAGGGTGGTATCAAGTGCCGAGT			
GEL3	**F:** CGTTGTCAGCGGAGGTATCGTC	PADG_04918	193 bp	Navarro et al., 2021
	**R:** AGGGCAGGTTCGGAGTTCAGTG			
KRE6	**F:** CGCAGTCTGTGAGATCTATTAC	PADG_07170	144 bp	This study
	**R:** GCGTCACTCCATACCAAA			

### Statistical analysis

Graphs and statistical analysis were performed using GraphPad Prism, version 5.0 (GraphPad Software, Inc., San Diego, CA). Results were presented as mean ± standard deviation and compared by analysis of variance (ANOVA) followed by the Bonferroni test. Statistical significance was considered when *p* < 0.05.

## Results

### Characterization of biofilms from different species of *Paracoccidioides* spp.

**Figure 1:**
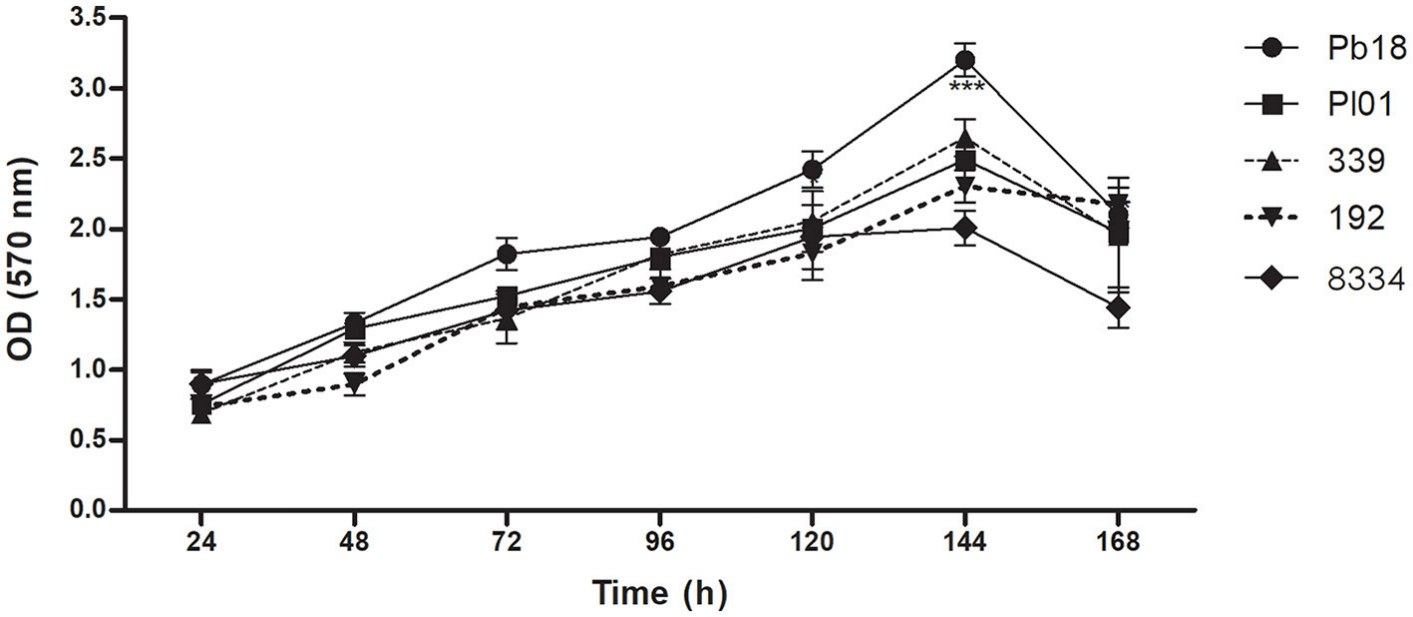
Quantification of the total biomass of *P. brasiliensis* (Pb18), *P. lutzii* (Pl01/8334), and *P. restrepiensis* (339/192) biofilms. Quantification of biomass of different biofilm *P. brasiliensis* strains by crystal violet methodology. Optical density reading at 570 nm of Pb18, Pl01, Pr339, Pr192, Pl8334 biomass. The results were expressed as the mean and standard error of the mean, and the experiments were carried out in six replicates and on three different occasions (*n* = 18). Two-way ANOVA. ***p < 0.001.

*P. brasiliensis* (Pb18), *P. lutzii* (01, 8334), and *P. restrepiensis* (339, 192) were able to form a biofilm, with large amounts of biomass production (Figure 1) increasing over time. According to the statistical analyses performed between the strains, there was a significant difference in biofilm formation at 120, 144, and 168 h for the Pb18 strain when compared with the Pl01, Pl8334, Pr339, and Pr192 strains, showing significant *p* < 0.05, *p* < 0.01, and *p* < 0.001, respectively. The maturation of the biofilms occurred in 144 h of incubation, observing a significant increase of the biomass in this time when compared to the times of 24, 48, 72, and 96 h (*p* < 0.001) and the decrease in the time of 168 h, which can be explained as the dispersion phase of biofilms.

**Figure 2:**
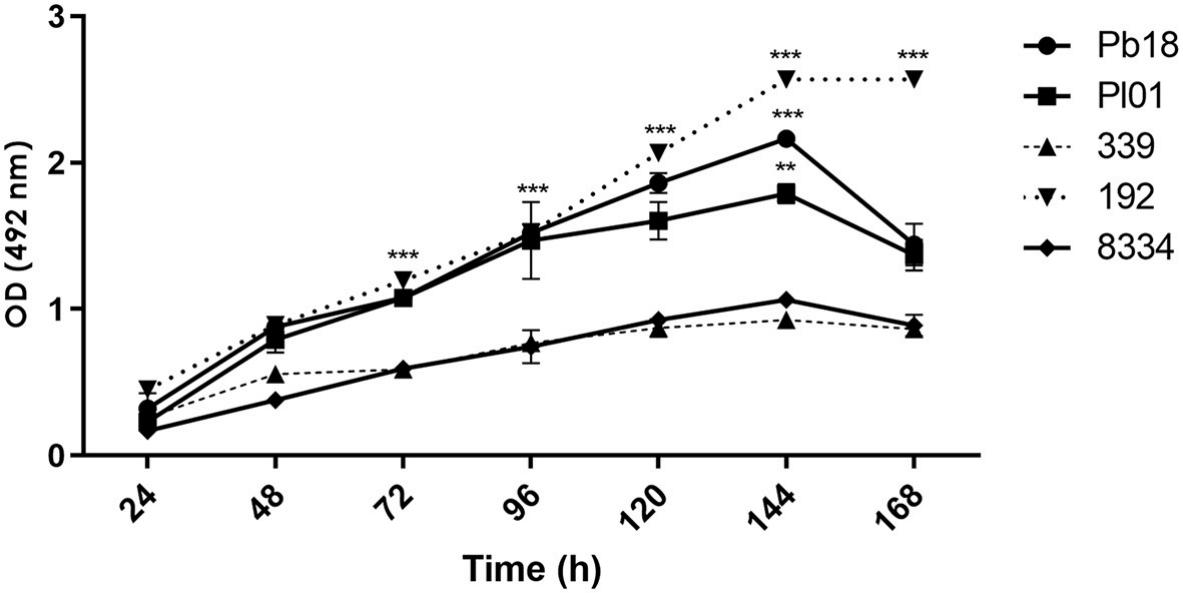
Quantification of metabolic activity of biofilms formed by *P. brasiliensis* (Pb18), *P. lutzii* (Pl01/8334), and *P. restrepiensis* (339/192) determined by XTT. Optical density reading at 492 nm. The results were expressed as the mean and standard error of the mean, and the experiments were carried out in six replicates and on three different occasions. Two-way ANOVA analysis. **p < 0.01 and ***p < 0.001.

*Paracoccidioides* is a slow-growing and demanding fungus that was initially assessed at 24 h. Previous studies indicated that biofilm formation before this time was nearly negligible. In the period between 24 and 120 h, this activity was significantly increased for all analyzed strains. At 144 h, the biomass and metabolic activity reached the maximum value for all strains, and there was a significant difference (*p* < 0.001) between them, except for strains Pr339 and Pb8334 (*p* > 0.05). After 144 h, readings were decreased, except for strain Pr192, where no changes were observed (Figure 2). After 168 h, the biofilms can enter the dispersion phase.

### Determination of the structure of the *Paracoccidioides* spp. biofilm through confocal laser scanning microscopy

In this step, only *P. brasiliensis* (Pb18) and *P. lutzii* (Pl01) strains were used due to the clinical importance of these species and their incidence in Brazil and other countries. The images obtained by confocal microscopy show the overlap of the three dyes and indicate the orthogonal section of the biofilm in 3D. The biofilms gradually increased their thickness over the analyzed times, up to the total maturation time (144 h). A cluster of yeast-like forms was observed, indicating the formation of microcolonies and an increase in thickness for both species as the incubation period progressed. Two points per well of biofilms were evaluated, and the images were edited in ImageJ 1.51p software and ZEN Support Software 4.1.

**Figure 3:**
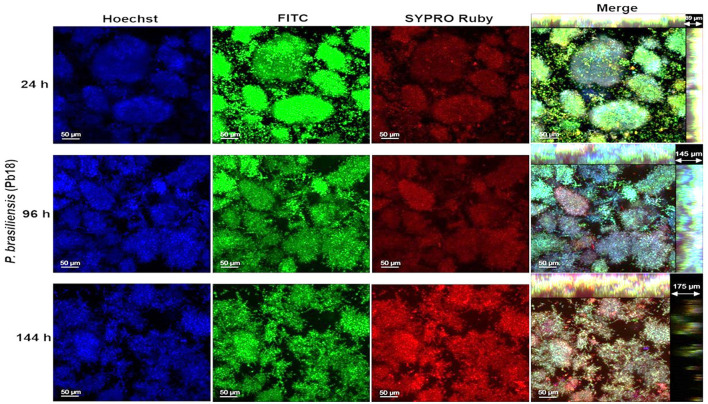
Representative confocal microscopy images of *P. brasiliensis* (Pb18) biofilms obtained by confocal microscopy (CLSM). Biofilm of *P. brasiliensis* observed in confocal microscopy (24–144 h). The nucleic content was marked with Hoechst (blue), the fungal cell walls with FITC (green), and the protein content with SYPRO Ruby (red) and merge-overlapping. The clusters characteristic of biofilms of this species (microcolonies) are observed.

**Figure 4:**
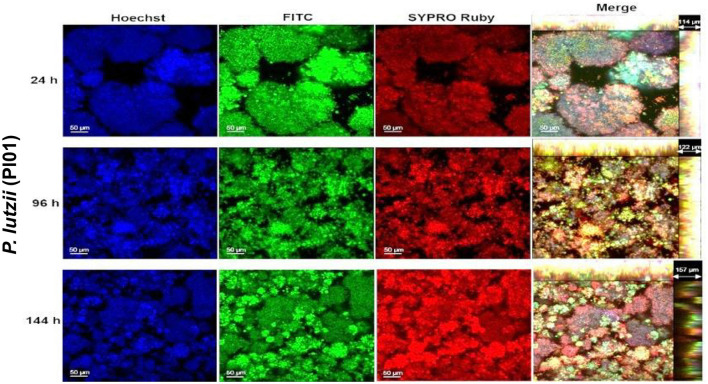
Representative confocal microscopy images of *P. lutzii* (Pl01) biofilms obtained by confocal microscopy (CLSM). Biofilm of *P. lutzii* observed in confocal microscopy (24–144 h). The nucleic content was marked with Hoechst (blue), the fungal cell walls with FITC (green), the protein content with SYPRO Ruby (red), and merge-overlapping. The clusters characteristic of biofilms of this species (microcolonies) are observed.

The protein content was more evident in the mature biofilm of *P. brasiliensis*, but in *P. lutzii*, the red coloration occurred more intensely from 96 h of biofilm formation (Figures 3, 4). Microcolonies of different sizes are observed, spaced on the surface and with little protein content in the matrix. At 96 h, the spacing between microcolonies is reduced compared to 24 h and is comparable to 144 h (Figure 3). This decrease in space may be due to the multiplication of cells in the microcolonies and the production of extracellular matrix components that promote their growth. However, from 96 to 144 h, there is an increase in the abundance of protein content in the extracellular matrix, which may have contributed to the increase in the thickness of these biofilms.

*P. lutzii* builds microcolonies much larger than those of *P. brasiliensis*. *P. lutzii* (Pl01) biofilm has a smaller thickness than that observed for *P. brasiliensis* (Pb18), and the increase over time was also less pronounced, as observed in Figure 4. The same was observed when the biomass was quantified by crystal violet (Figure 1). Furthermore, the distribution and size of microcolonies are quite different between the two strains, which may influence how these species respond to therapeutic interventions.

### Determination of extracellular matrix components of *P. brasiliensis* and *P. lutzii* biofilms

**Figure 5:**
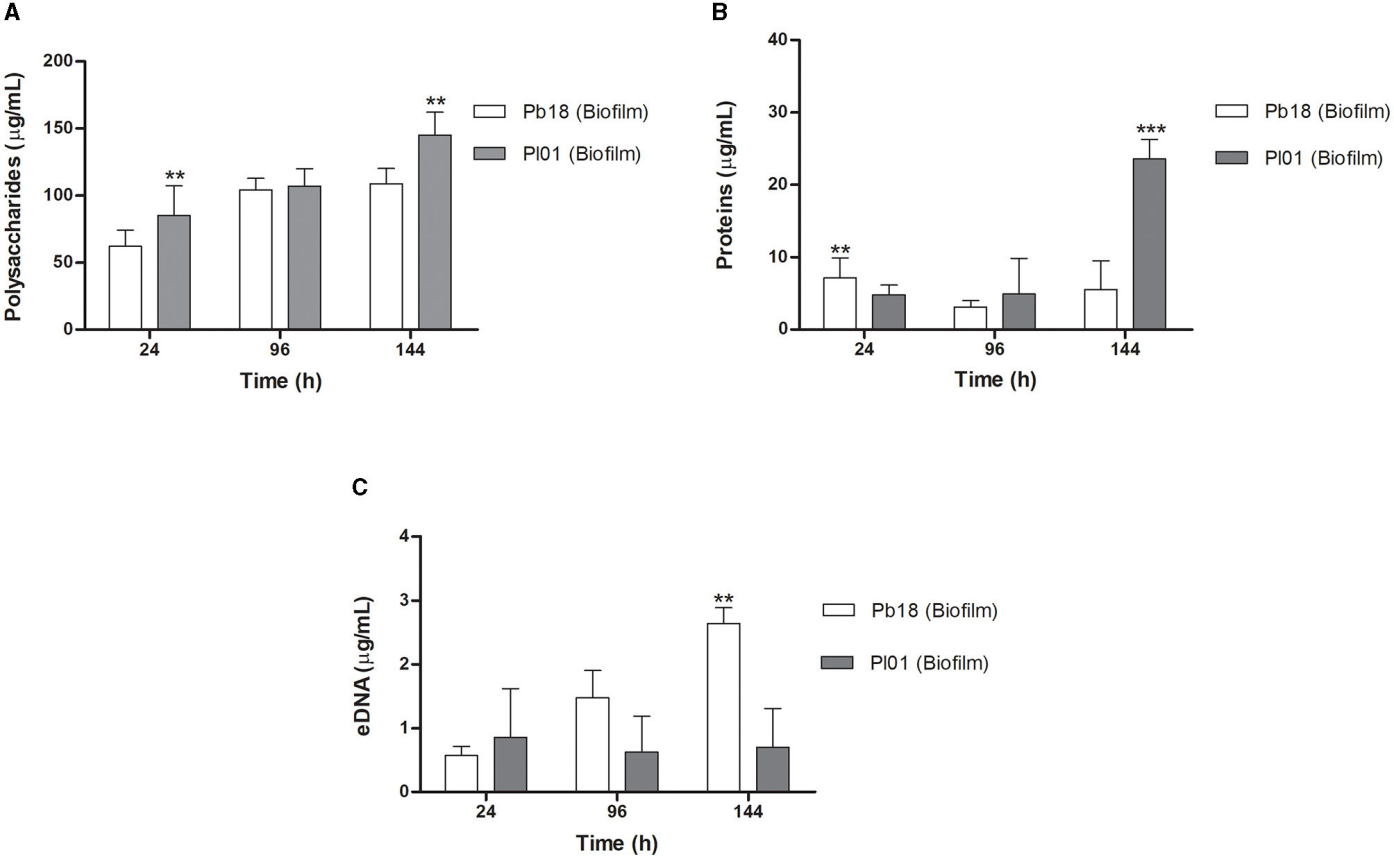
Results of polysaccharides, proteins, and extracellular DNA quantity of *P. brasiliensis* (Pb18) and *P. lutzii* (Pl01) biofilms. **(A)** Amount of total polysaccharides measured by the Dubois method. **(B)** Amount of total proteins measured by the Bradford method, and **(C)** the amount of DNA evaluated by the phenol:chloroform: isoamyl alcohol method. The polysaccharide content was calculated using glucose for the standard curve (0–200 μg/mL) and bovine serum albumin protein (BSA). Data expressed as mean and standard deviation performed in duplicate on three different occasions using two-way ANOVA analyses. **p < 0.01 and ***p < 0.001.

Quantitative data of polysaccharides, proteins, and eDNA from biofilms of *P. brasiliensi*s (Pb18) and *P. lutzii* (Pl01) are shown in Figure 5. In the first 24 h, *P. lutzii* (Pl01) biofilm produced more polysaccharides (*p* < 0.01) than *P. brasiliensis* (Pb18) biofilm, as shown in Figure 5A. In the period of 96 h, there was no significant difference in the production of polysaccharides by Pb18 or Pl01 (*p* > 0.05). In 144 h, Pl01 produced more polysaccharides than Pb18 (*p* < 0.01).

*P. brasiliensis* (Pb18) showed higher protein production at 24 h, with a slight decline at 96 h and, at 144 h, comparable to the 24 h period (*p* < 0.01). *P. lutzii* (Pl01) did not show a significant increase in the period from 24 to 96 h (*p* > 0.05), but a significant increase in protein production at 144 h compared to the production at 24 and 96 h (*p* < 0.001) (Figure 5B). This data agrees with the confocal microscopy results (Figure 4).

*P. brasiliensis* (Pb18) showed significant eDNA production during the biofilm maturation (*p* < 0.01). *P. lutzii* (Pl01) produced eDNA in the first 24 h and a slight increase at 144 h (Figure 5C).

Among the evaluated components, polysaccharides are responsible for ~96.9 and 96.2% of the matrix of *P. brasiliensis* and *P. lutzii*, respectively, corresponding to the highest amount concerning the other components. Proteins are responsible for 2.7 and 3.5% of the matrix of *P. brasiliensi*s and *P. lutzii*, respectively. Another key component of the matrix is eDNA. In the biofilm matrix of *P. brasiliensis*, a small amount of eDNA of approximately 0.3% was verified, and 0.4% for *P. lutzii*. When comparing the two species of *Paracoccidioides*, concerning the components of the extracellular matrix of the biofilm, we did not observe a significant difference in the values.

### qRT-PCR standardization

The mean ratio value of A260/280 for all RNA samples was 2.1–2.2, reflecting high purity (>1.8 to A260/280 nm), and the integrity of RNA was verified by electrophoresis (data not shown). Real-time PCR standardization was performed to determine the primer concentration as well as the efficiency of amplification. We calculated the slope for each primer pair and found values near the ideal, −3.2, efficiencies ranging from 90.99 to 105.1%, and an *R*^2^ value of 0.99, meeting the requirement for qRT-PCR (Table 2). Primers were tested for a non-specific product by amplicon separation on agarose gel electrophoresis and size confirmation (ranging from 118 to 193 bp according to the gene) at the end of a qPCR run as well as the analysis of melting curves that show only one peak for all samples indicating the amplification of a single product (Supplementary Figures S1A, B). The best endogenous control was the L34 gene, which showed lower expression variation between the samples and was used for the qRT-PCR analysis.

**Table 2:** Oligonucleotides used in qPCR and its respective amplification efficiencies, correlation coefficients (*R*^2^)^‡^, and slope values.

**Oligonucleotide**	**Slope**	**Efficiency**	***R* ^2^**
L34	−3.276	101.9%	0.9938
ACT1	−3.238	103.62%	0.9973
ENOLASE	−3.251	103.04%	0.9979
14-3-3	−3.291	101.2%	0.9983
FKS1	−3.253	105.1%	0.9986
AGS1	−3.355	98.63%	0.9942
GEL3	−3.548	91.34%	0.9968
KRE6	−3.558	90.99%	0.9944

^‡^R^2^, correlation coefficient.

### Modulation of expression of genes related to the synthesis of cell wall component glucans and its delivery and adhesins of *P. brasiliensis* growth as a biofilm

**Figure 6:**
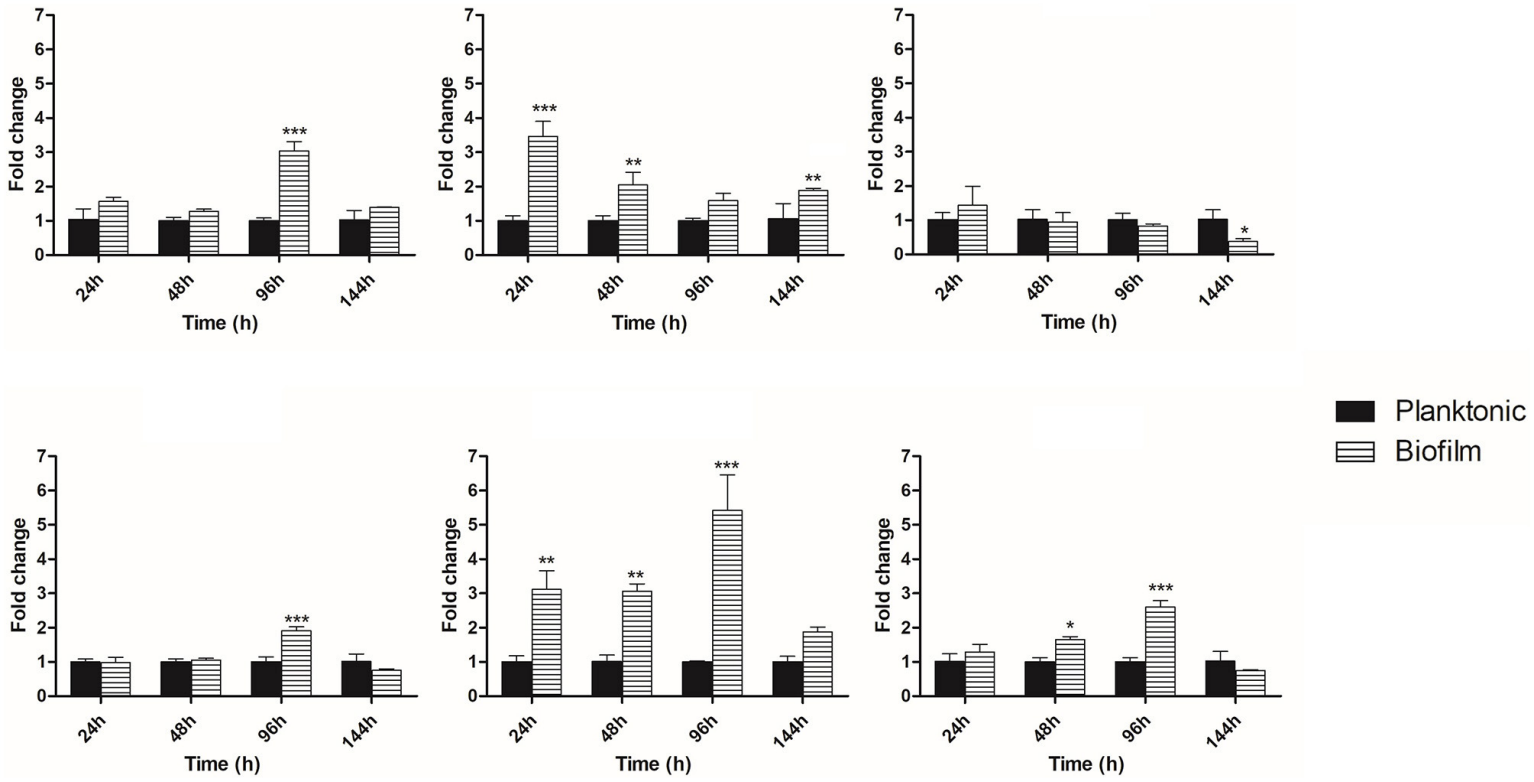
Temporal analysis of expression of genes related to synthesis and maintenance of cell wall components and adhesins. Differential expression of genes in biofilm condition when compared to the planktonic growth at 24, 48, 96, and 144 h. Genes were evaluated by qRT-PCR. Conditions were compared using two-way ANOVA followed by a Bonferroni post-test. ***p < 0.0001; **p < 0.01; *p < 0.05. FKS1, 1,3-β-glucan synthase; AGS1, 1,3-α-glucan synthase; KRE6, glycosyl hydrolase required for 1,6-β-glucan synthesis; GEL3, 1,3-β-glucanosyltransferase, enolase and 14-3-3 genes.

**Figure 7:**
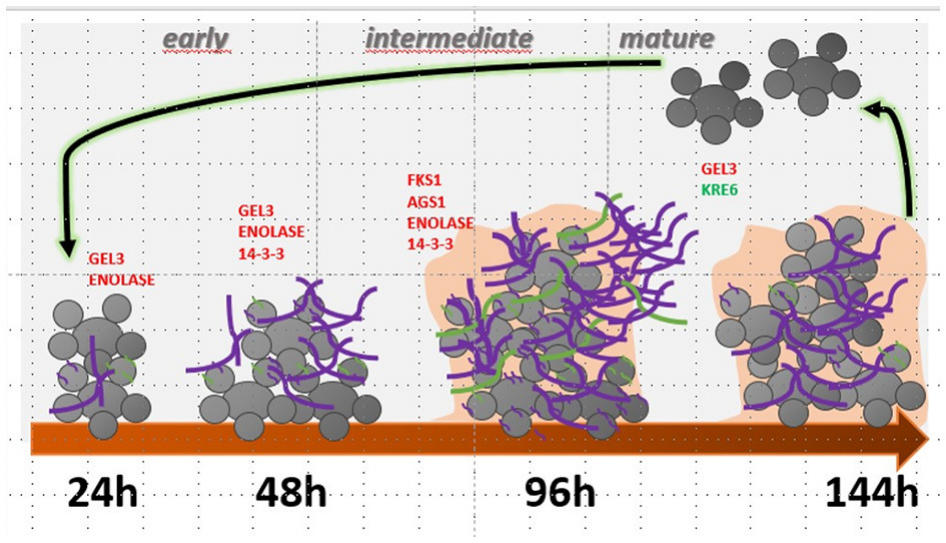
Summary of gene modulation during the formation and maturation stages of *P. brasiliensis* biofilm. In red, genes are significantly more expressed, and in green, with reduced expression. 1,3-β glucan is highlighted in purple, and α-glucan in green.

Quantitative PCR was performed to analyze the expression of some genes involved in *P. brasiliensis* adhesion and the synthesis and maintenance of glucans to assess whether they would be altered during the biofilm condition. During the early phase of biofilm formation (24 h), the genes GEL3 and enolase were up-regulated, with an increased expression of 3.4- and 3.1-fold compared to the planktonic cells. Posteriorly, at 48 h of biofilm maturation, GEL3, enolase, and 14-3-3 had their expression increased 2-, 3-, and 1.6-fold, respectively. At the intermediate time of biofilm maturation (96 h), we observed the most significant increases in several genes, such as FKS1 (3.4-fold), AGS1 (1.9-fold), enolase (5.4-fold), and 14-3-3 (2.6-fold). Finally, at 144 h, considered the stage of mature biofilm, a decreased expression of KRE6 (2.7-fold) and increased expression of GEL3 (1.8-fold) was found (Figure 6). The results of qRT-PCR are summarized in Figure 7.

## Discussion

In this work, we analyze biofilm formation in different species of *Paracoccidioides*. The ideal time for the biofilm maturation of most studied strains was 144 h, a result compatible with those described by Sardi et al. (2015) for *P.brasiliensis* biofilm. The new classification concerning the genus *Paracoccidioides* includes five species (Carrero et al., 2008; Hrycyk et al., 2018), but as can be seen in the crystal violet and XTT assays, the studied strains of three different species have behavior similar in terms of ability to form a biofilm (biomass and metabolic activity).

According to the literature, biofilms share several common characteristics, such as the production of extracellular polymeric substances. The composition of the extracellular matrix varies according to the microorganism and can be affected by the environment and changes in growth conditions, such as the amount of nutrients, temperature, and pH. Zarnowski et al. (2014) reported that the *C. albicans* biofilm matrix comprises 55% protein, 25% carbohydrates, 15% lipids, and 5% nucleic acid. For *Aspergillus fumigatus*, the biofilm matrix contains ~40% proteins, 43% polysaccharides, and at least 8% lipids (Reichhardt et al., 2015). For *C. albicans*, eDNA was reported to contribute to biofilms' structural integrity and role in antifungal resistance (Martins et al., 2010; Sapaar et al., 2014).

The matrix is self-produced in the extracellular environment, from incorporating diverse community products to creating a unique structure (Mitchell et al., 2015). The exact composition of the ECM of *P. brasiliensis* biofilm and the regulatory mechanisms of biofilm formation are still unclear. Here, variations in the amount of polysaccharides, proteins, and extracellular DNA (eDNA) were demonstrated after ECM extraction of *P. brasiliensis* and *P. lutzii* biofilms. Increased polysaccharide production at different stages of the *in vitro* biofilm (24, 96, and 144 h) was observed, and the same with total proteins and eDNA. Differences between the two species of *Paracoccidioides* were detected in biofilm growth.

Studies carried out with *Candida* have shown an alternation in the amount of polysaccharides and proteins in the biofilm matrix of different species of this genus (Silva et al., 2009). Generally, *C. parapsilosis* biofilm matrices had large amounts of carbohydrates and relatively smaller amounts of proteins. In contrast, biofilm matrices of *C. tropicalis* strains showed lower concentrations of proteins and carbohydrates, and *C. glabrata* biofilm matrices had relatively higher amounts of protein and carbohydrates than other species (Silva et al., 2009).

Polysaccharides are key constituents of the ECM of fungal biofilms. The polysaccharide 1,3-β-glucan is present in *Candida albicans* biofilm, associated with biofilm protection against antifungal agents (Taff et al., 2012). Additionally, they act as “scaffolds” for connecting other biomolecules, playing an essential role in the maintenance and function of biofilms (Sheppard and Howell, 2016). Therefore, the present study evaluated the genes' expression in controlling matrix delivery and enzymes related to carbohydrate production and modification to be incorporated into the ECM.

The 1,3-β-glucan synthase (Fks1) is required for 1,3-β-glucan production and biofilm matrix development in fungi and can also mention enzymes involved in its modification and delivery and/or excretion to the ECM matrix, as the 1,3-β-glucanosyltransferase (Gel3) (Douglas et al., 1997; Nett et al., 2007; Massey et al., 2023). The FKS1 gene of *P. brasiliensis* showed an increased expression throughout the maturation of biofilm, which was more expressive at 96 h (intermediate phase), with a 3.4-fold increase when compared to the planktonic culture, suggesting that this polysaccharide can be a component of the ECM of *P. brasiliensis* biofilm that coincides with the maturation phase with the most significant thickening of ECM.

Evidence suggests that Gel3, a glucan elongation protein, has 1,3-β-glucanosyltransferase activity, acting at the cross-linking of cell wall components in fungi, and therefore associated with cell wall morphogenesis (Popolo and Vai, 1999; Mouyna et al., 2000). In *A. fumigatus*, AfGel1p catalyzes the 1,3-β-glucanosyltransferase reaction *in vitro* in two steps: (1) a glycosidic bond of a 1,3-β-glucan donor is cleaved, and the reduced moiety is released, (2) then the new reduced end is transferred to a non-reducing end of a 1,3-β-glucan acceptor chair. Therefore, forming a new bond between the 1,3-β-glucan donor and acceptor results in the 1,3-β-glucan elongation at branch points of other glucans and also creates anchorage points for mannoproteins, chitin, and galactomannan. Thus, 1,3-β-glucanosyltransferase acts similarly to glycosidic hydrolases in the first step but as a carbohydrate in the second, being preferable to a water molecule (Mouyna et al., 2005; Ragni et al., 2007).

Furthermore, we have α-glucan as the main polysaccharide (95%) located in the outermost layer of the yeast form of *P. brasiliensis*; its presence is correlated with virulence, possibly due to the masking of β-glucan, which leads to a decrease in the recognition of the immune system (Souza et al., 2019). Among the genes involved in the α-glucan synthesis to *P. brasiliensis*, we have the AGS1, in which we observed a 1.9-fold increase in expression at 96 h of biofilm growth, and perhaps this polysaccharide can be part of the ECM component produced by the biofilm of this pathogen. In *Aspergillus fumigatus*, the presence of α-glucan in the ECM has already been described (Beauvais et al., 2007). The biofilm present in the aspergilloma produces a thicker layer of ECM containing α-glucan, which is absent in biofilms formed in invasive aspergillosis (Fontaine et al., 2010; Loussert et al., 2010).

Gel(s) are proteins homologous to glycophospholipid anchoring (Gas) proteins, pH-regulated (Phr), and essential to the development of pseudohyphae protein (Epd), which are present in different fungal species belonging to the GH72 (glycosyl hydrolase 72) family cluster (Saporito-Irwin et al., 1995; Mühlschlegel and Fonzi, 1997; Nakazawa et al., 1998; Mouyna et al., 2000; Ragni et al., 2007). The GEL3 expression in *P. brasiliensis*, as well as the amount of the respective protein, is increased in the mycelial phase, suffering a drastic reduction after 24 h when the temperature changes from 22 to 36°C, reaching low levels in the yeast phase, that corroborates with the predominance of 1,3-β glucan in the mycelium phase of this fungus (da Silva Castro et al., 2009). Additionally, *P. brasiliensis* GEL3 can partially rescue the GAS1 mutation in *S. cerevisiae* (da Silva Castro et al., 2009). The respective mutation leads to reduced growth phenotypes, morphological alterations, sensitivity to calcofluor, and increased cell wall permeability (Popolo and Vai, 1999; Vai et al., 2000). In the present study, we observed that GEL3 is more expressed at all evaluated times of biofilm maturation, mainly at 24, 48, and 144 h.

Gas1 (*Saccharomyces cerevisiae*), Phr1 and Phr2 (*C. albicans*), Gel1 and Gel2 (*A. fumigatus*) act actively in the biosynthesis and morphogenesis through the correct incorporation of glucan molecules in the cell wall being also related to the virulence of *C. albicans* and *A. fumigatus* in a murine model of infection (Ghannoum et al., 1995; Mouyna et al., 2005). PHR1 expression is increased in the biofilm vs. planktonic culture, and its deletion reduces the glucan in *C. albicans* (Murillo et al., 2005; Nett et al., 2009). In contrast, another study showed that the overexpression of PHR2, but not PHR1, increased biofilm occupancy but not adherence or biomass (Cabral et al., 2014). Such differences can reflect variations related to the medium composition and pH used for biofilm formation and the implication of these enzymes in this process (Popolo et al., 2017).

In the present study, the expression of GEL3 is increased. On the contrary, we have a greater expression of FKS1 and a decrease in the expression of GEL3 at 96 h, suggesting that in *P. brasiliensis* there is a feedback signaling for the fungus to produce additional 1,3-β glucan during biofilm growth when production occurs of enzymes necessary for its modification and delivery to the ECM is reduced. Previously, Taff et al. (2012) correlated the expression level of enzymes related to 1,3 β-glucan synthesis and glucan modification to their delivery to ECM and maturation. Mutants for glucan-modifying enzymes, which have reduced glucan delivery to the extracellular space, signal the fungus to undergo increased 1,3-β glucan synthesis.

In addition to glucan synthesis, Kre6 is a glycosyl hydrolase associated with 1,6-β-glucan synthesis, and its deletion in *C. albicans* did not impact biofilm formation (Kurita et al., 2011). In *P. brasiliensis* the KRE6 behavior was not like GEL3. KRE6 has a slight increase at 24 h, but at later times of biofilm maturation (48, 96, and 144 h), its expression was lower when compared to planktonic cells, being more evident at 144 h, possibly this polysaccharide is also present in lower amounts in the ECM to this microorganism. In yeast cells of *P. brasiliensis* we have 95% of glucan and 5% of β-glucan in both anomeric forms, predominantly 1,3-β-glucan and too little of 1,6-β-glucan (Kanetsuna et al., 1972).

Other genes evaluated in this study refer to adhesins. Adhesion is the first step to biofilm formation and pathogenesis, so this process underlies many consequences for fungal lifestyle, commensalism, and infection. Adhesion to several substrates can occur through different interactions, such as hydrophobic interactions, electrostatic attraction, and specific ligands called adhesins (Chaffin et al., 1998; Chandra et al., 2001; Jeng et al., 2005; Ramage et al., 2005).

Adhesins on the fungal cell surface are often identified as downstream effectors in biofilm proteomic and transcriptomics studies. Once the biofilm is formed, there is a progression from one adhesin to another, and we have limited knowledge regarding the consequences of its expression changes. A related question would be the role of external signaling: how does a cell know it is “stuck” to a substrate or a neighboring cell? In this sense, we have the quorum-sensing acting, but once immobilized, the cells act differently from planktonic cells, and the adhesion itself must be a determinant for this (Lipke, 2018).

In our qRT-PCR analysis, we evaluated the expression of two well-described adhesins for *P. brasiliensis*, enolase and 14-3-3 (Donofrio et al., 2009; Marcos et al., 2012, 2016; da Silva et al., 2013; de Oliveira et al., 2015). Enolase (ENO) is an enzyme of the glycolytic pathway considered also known to be a moonlighting protein in *P. brasiliensis* (Marcos et al., 2014) as well as for other pathogens (Fox and Smulian, 2001; Jong et al., 2003). Previously, Sardi et al. (2015) evaluated the expression of some adhesins and hydrolytic enzymes in mature biofilm condition (144 h) of *P. brasiliensis*, under low oxygen tension and culturing the fungi in mFUM medium (Modified Fluid Universal Medium) and observed 1.5-fold increase to enolase expression. Here, we included initial and intermediate times of biofilm formation and BHI as a medium and observed a considerable increase of expression at 24, 48, and 96 h, and to 144 h, this increase was not so expressive (1.8-fold).

These data may suggest that enolase is more important in the initial phases (probably in adhesion) of *P. brasiliensis* biofilm formation or changes in the metabolic process required by biofilm formation. The increase in the assimilation of carbohydrates via glycolysis with the production of a pool of pyruvate and amino acids is necessary for the increase in biomass that requires greater energy availability. Once the phase is reached, there is a decrease in the expression of related genes, resulting in a decrease in metabolic activity or stationary phase of the biofilm, as demonstrated in *C. albicans* (Yeater et al., 2007).

*C. albicans* is capable of secreting enolase, which is detected in the ECM of the biofilm (Silva et al., 2014); it can be re-adsorbed on the cell surface through ALS3 interaction, acting as a “bridge” for the interaction of the latter with host proteins (plasminogen and kininogen) (Karkowska-Kuleta et al., 2021) and also support for polymicrobial interaction in biofilms (Bartnicka et al., 2019).

Widely distributed among eukaryotes, 14-3-3 proteins are highly conserved and ubiquitous, with subunits of approximately 30 kDa, showing multiple isoforms. In the present study, we observed an increased expression of 14-3-3 at initial and intermediate times. This higher expression can probably contribute to changing the expression of other factors related to fungal adhesion with consequent ECM increase and biofilm formation; once biofilm is consolidated, its expression is reduced.

Several studies demonstrate that 14-3-3 proteins act as regulators of multiple physiological processes, signal transduction, primary metabolism, cell cycle regulation, protein trafficking, and reactions to abiotic and biotic stress; due to its lack of catalytic activity, many of its functions occur through the binding to other proteins (Shi et al., 2019). In *P. brasiliensis*, 14-3-3 seems involved in fungal dimorphism (Marcos et al., 2016). Previously, Carlton (2018) demonstrated that the isolate with low expression of 14-3-3 has more significant metabolic activity during biofilm growth, unlike the planktonic form, suggesting that the biofilm growth condition presents an altered state of metabolism (Borriello et al., 2006; Nguyen et al., 2011). Sardi et al. (2024) showed that the isolate silenced for 14-3-3 presents a reduced expression of other genes in planktonic or biofilm growth conditions, such as enolase, gp43, and GAPDH, as well as produces biofilm with a lower amount of ECM. Then, 14-3-3 is known to have multiple functions and can also act in the gene expression modulation of virulence factors and adhesin and influence the biogenesis of ECM directly or indirectly.

## Conclusion

In conclusion, this study highlights the potential for biofilm formation by three species of *Paracoccidioides* and the main components of the extracellular matrix that may contribute to pathogenicity, mainly through its roles in promoting antifungal drug tolerance and immune evasion. The pathway related to the synthesis and delivery of polysaccharides, constituents of the extracellular matrix, considered the key to differentiating biofilm and planktonic growth conditions, is temporally modulated along the biofilm kinetics of formation in *P. brasiliensis*, and has a greater expression of important multifunctional proteins mainly involved in the adhesion at early stages and biofilm initiation. In the future, there is a need to study the composition of the extracellular matrix of the *P. brasiliensis* biofilm and isolates with low expression of the elucidated targets to deepen the knowledge about their fundamental importance and whether they can be considered attractive for the development of new therapies targeting their inhibition to decrease biofilm formation or even as biofilm markers.

## Data availability statement

The original contributions presented in the study are included in the article/Supplementary material, further inquiries can be directed to the corresponding author.

## Author contributions

LO: Formal analysis, Investigation, Software, Writing—original draft. CM: Data curation, Formal analysis, Investigation, Software, Validation, Writing—original draft, Writing—review & editing. AC: Conceptualization, Visualization, Writing—review & editing. KM-A: Conceptualization, Supervision, Writing—review & editing. RP: Conceptualization, Supervision, Visualization, Writing—original draft. AF-A: Data curation, Methodology, Supervision, Visualization, Writing—review & editing. MM-G: Conceptualization, Data curation, Funding acquisition, Methodology, Project administration, Resources, Supervision, Visualization, Writing—original draft, Writing—review & editing.

## Supplementary material

Supplementary Figure S1 Specific amplification at the end of the qPCR run. **(A)** Melt curve analysis of each primer pair showing a single peak. **(B)** Agarose electrophoresis gel of amplicons demonstrating a single band with correspondent size according to the gene. MW, molecular weight.
