# Supplementary figures and images for: Paracoccidioides spp.: the structural characterization of extracellular matrix, expression of glucan synthesis and associated genes and adhesins during biofilm formation
Source: Front Microbiol. 2024 Mar 7;15:1354140. doi: 10.3389/fmicb.2024.1354140 (PMC10955377; doi:10.3389/fmicb.2024.1354140)

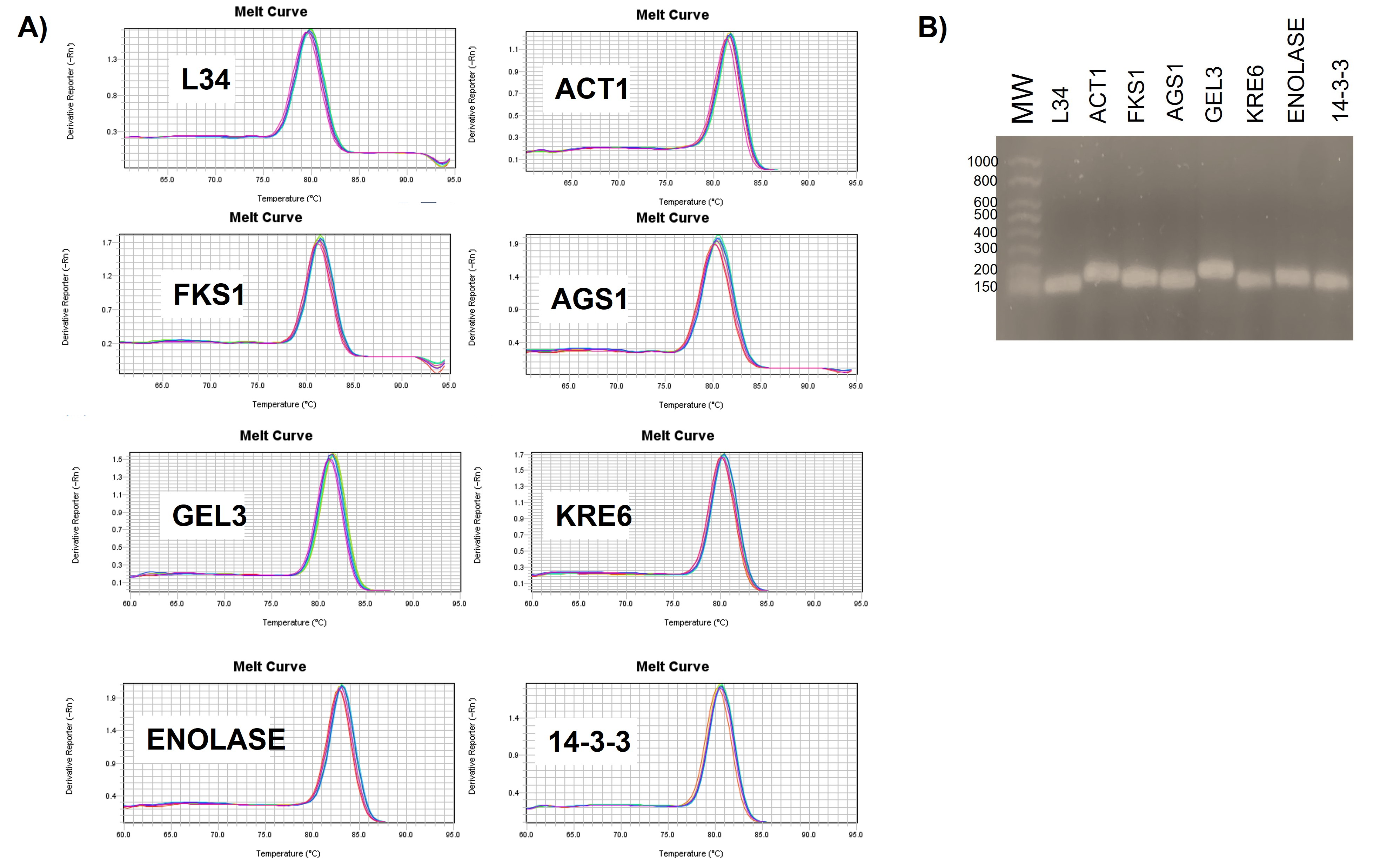

Supplement: Supplementary Figure S1 — Specific amplification at the end of the qPCR run. (A) Melt curve analysis of each primer pair showing a single peak. (B) Agarose electrophoresis gel of amplicons demonstrating a single band with correspondent size according to the gene. MW, molecular weight. [file Image_1.TIFF]
